# Exploring the Importance of Race and Gender Concordance Between Patients and Physical Therapists in Digital Rehabilitation for Musculoskeletal Conditions: Observational, Longitudinal Study

**DOI:** 10.2196/65354

**Published:** 2024-10-29

**Authors:** Anabela C Areias, Dora Janela, Maria Molinos, Virgílio Bento, Carolina Moreira, Vijay Yanamadala, Steven P Cohen, Fernando Dias Correia, Fabíola Costa

**Affiliations:** 1 Sword Health, Inc Draper, UT United States; 2 Instituto de Ciências Biomédicas Abel Salazar Porto Portugal; 3 Department of Surgery Quinnipiac University Frank H Netter School of Medicine Hamden, CT United States; 4 Department of Neurosurgery Hartford Healthcare Medical Group Westport, CT United States; 5 Northwestern Feinberg School of Medicine Chicago, IL United States; 6 Walter Reed National Military Medical Center Uniformed Services University of the Health Sciences Bethesda, MD United States; 7 Neurology Department Centro Hospitalar e Universitário do Porto Porto Portugal

**Keywords:** musculoskeletal pain, physical therapy, telerehabilitation, eHealth, racial/ethnic concordance, patient–provider concordance, physical therapy, digital rehabilitation, musculoskeletal conditions

## Abstract

**Background:**

Race/ethnicity and gender concordance between patients and providers is a potential strategy to improve health care interventions. In digital health, where human interactions occur both synchronously and asynchronously, the effect of concordance between patients and providers is unknown.

**Objective:**

This study aimed to evaluate the impact of race/ethnicity or gender concordance between patients and physical therapists (PTs) in engagement and the clinical outcomes following a digital care program (DCP) in patients with musculoskeletal (MSK) conditions.

**Methods:**

This secondary analysis of 2 prospective longitudinal studies (originally focused on assessing the acceptance, engagement, and clinical outcomes after a remote DCP) examined the impact of both race/ethnicity concordance and gender concordance between patients and PTs on outcomes for a digital intervention for MSK conditions. Outcomes included engagement (measured by the completion rate and communication, assessed by text interactions), satisfaction, and clinical outcomes (response rate, ie, percentage of patients achieving at least a minimal clinically important change in pain, measured by the Numerical Pain Rating Scale [NPRS]; anxiety, measured by the Generalized Anxiety Disorder 7-item scale [GAD-7]; depression, measured by the Patient Health Questionnaire 9-item [PHQ-9]; and daily activity impairment, measured by the Work Productivity and Activity Impairment [WPAI] questionnaire).

**Results:**

Of 71,201 patients, 63.9% (n=45,507) were matched with their PT in terms of race/ethnicity, while 61.2% (n=43,560) were matched for gender. Concordant dyads showed a higher completion rate among White (adjusted odds ratio [aOR] 1.11, 95% CI 1.05-1.19, *P*<.001) and Hispanic (aOR 1.27, 95% CI 1.08-1.54, *P*=.009) groups, as well as women (aOR 1.10, 95% CI 1.06-1.18, *P*<.001), when compared to discordant dyads. High and similar levels of interaction between patients and PTs were observed across race/ethnicity and gender dyads, except for Asian concordant dyads (adjusted β coefficient 5.32, 95% CI 3.28-7.36, *P*<.001). Concordance did not affect satisfaction, with high values (>8.52, 95% CI 8.27-8.77) reported across all dyads. Response rates for pain, anxiety, and daily activity impairment were unaffected by race/ethnicity concordance. An exception was observed for depression, with White patients reporting a higher response rate when matched with PTs from other races/ethnicities (aOR 1.20, 95% CI 1.02-1.39, *P*=.02). In terms of gender, men had a slightly higher pain response rate in discordant dyads (aOR 1.08, 95% CI 1.01-1.15, *P*=.03) and a higher depression response rate in concordant dyads (aOR 1.23, 95% CI 1.05-1.47, *P*=.01).

**Conclusions:**

Race/ethnicity and gender concordance between patients and PTs does not translate into higher satisfaction or improvement for most clinical outcomes, aside from a positive effect on treatment completion. These results highlight the importance of other PT characteristics, in addition to race/ethnicity or gender concordance, suggesting the potential benefit of experience, languages spoken, and cultural safety training as ways to optimize care.

**Trial Registration:**

ClinicalTrials.gov NCT04092946, NCT05417685; https://clinicaltrials.gov/study/NCT05417685, https://clinicaltrials.gov/study/NCT04092946

## Introduction

Individual characteristics, including race and ethnicity, are recognized as major determinants of health according to the World Health Organization. Health disparities among racial and ethnic minorities are well documented, revealing decreased health care access, poorer health status, and prognosis among these populations [1-3].

Although future projections forecast an increase in population and diversity in the United States [4], diversity within the medical community is not growing at the same pace [5]. This imbalance has raised concerns about the ability to provide culturally competent care and address the specific needs of these patients, when the majority of the workforce is White [6]. As such, the concept of concordance, when a patient and a clinician have the same race/ethnicity or gender, has been explored to understand its potential impact on engagement and clinical outcomes.

Previous studies, predominantly conducted in primary care and psychology settings, have focused on understanding patients’ perspectives regarding clinicians’ demographics and their impact on therapeutic alliance, communication quality, and overall satisfaction. Findings from these studies are varied: some suggest that patient-clinician racial/ethnic or gender concordance offers benefits [7-11], while others do not [12-14]. Moreover, a recent systematic review studying the association between racial, gender, and multifactorial concordance reported inconclusive results on patient outcomes, advocating for further research [15]. This heterogeneity might be explained by singularities in the population, conditions, and settings under study.

In digital health, where care can be delivered both synchronously and asynchronously, the impact of concordance between patients and providers remains largely unexplored, particularly in the field of physical therapy. Digital rehabilitation has arisen as a solution to address geographical and time barriers, as well as clinician shortages. Previous research supports its effectiveness among several musculoskeletal (MSK) conditions [16-18] regardless of race/ethnicity [19] and socioeconomic backgrounds [20]. Given the rising adoption of digital rehabilitation programs, and the unique dynamics they introduce to the patient-therapist relationship (eg, duration and frequency of contacts, communication channels used), it is important to assess the association between concordance, engagement, and patient outcomes.

This study aimed to evaluate the impact of race/ethnicity or gender concordance between patients and physical therapists (PT) in engagement and the clinical outcomes following an asynchronous digital care program (DCP) in patients with MSK conditions in a real-world setting. Additional insights into this topic can enable providers and constituents to develop strategies that consider these aspects.

## Methods

### Study Design

This was a secondary analysis of 2 prospective, longitudinal, single-arm investigations focusing on the acceptability, feasibility, engagement, and clinical outcomes of a remote DCP for a population with acute and chronic MSK conditions [20-24]. The overarching aim of the original protocol also included the exploration of associations between the outcomes and patients’ demographic and clinical profiles. Patients participated in this study between January 1, 2022, and December 15, 2023. This study was reported in accordance with STROBE (Strengthening the Reporting of Observational Studies in Epidemiology) guidelines (Table S1 in Multimedia Appendix 1).

### Study Population

Patients, who were beneficiaries of employer health plans, reporting MSK pain in any of the following areas were included in accordance with eligibility criteria in the original investigations: ankle, elbow, hip, knee, low back, neck, shoulder, or wrist/hand. Patients self-reported their race/ethnicity in accordance with 2020 U.S. Census Bureau categories by the U.S. Office of Management and Budget (OMB) [25] as non-Hispanic Asian (hereafter referred to as Asians), non-Hispanic Black or African Americans (hereafter referred to as Black), Hispanic or Latino (hereafter referred to as Hispanic), non-Hispanic White (hereafter referred to as White), American Indian or Alaska Native, and Native Hawaiian or Other Pacific Islander.

Exclusion criteria defined in the original investigations were (1) health conditions incompatible with >20 minutes of light-to-moderate exercise; (2) ongoing cancer treatment; (3) the presence of red flags, defined as signs or symptoms suggestive of serious pathology (eg, rapid progressive motor weakness or sensory alterations, or bowel or bladder dysfunction), if uncleared by a physician; and (4) missing patient or PT gender or race/ethnicity. Additional exclusion criteria specific for this secondary analysis were patient race/ethnicity from American Indian or Alaska Native and Native Hawaiian or Other Pacific Islander, given the small sample size of these subgroups that precluded statistical analyses. Patients who were compliant with the intervention but missed a given reassessment survey were included.

### Intervention

This completely remote DCP, developed according to current guidelines [26-28], consisted of home-based exercise, education, and cognitive behavioral therapy (CBT) for up to 12 weeks (at least 3 exercise sessions per week were recommended), as described elsewhere [19,20].

**Figure 1:**
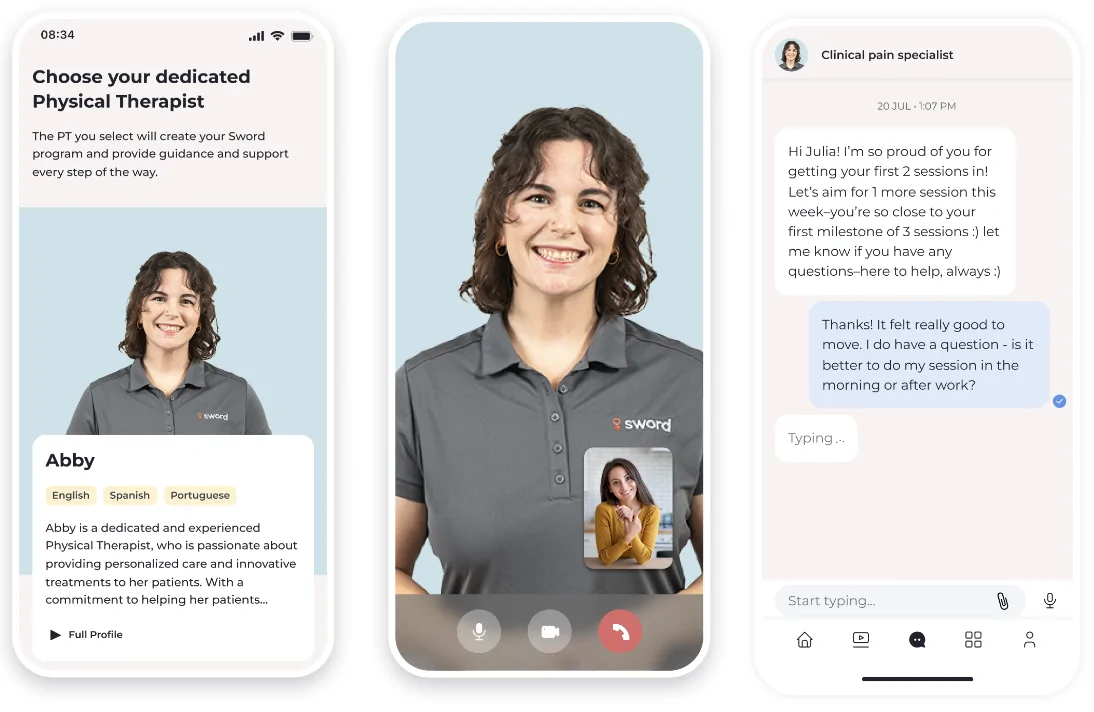
Screenshots of the patient app showcasing the content offered upon onboarding PT selection, during video calls, and during text messaging along the DCP (no real patients are portrayed in this figure). DCP: digital care program; PT: physical therapist.

Briefly, after enrollment, patients completed a baseline form on a dedicated website collecting demographic and clinical information. At the end of this form, patients could choose from a pool of available PTs presented with a photo and a mini biosketch (Figure 1). PTs were diverse in gender, race/ethnicity (Figure S2 in Multimedia Appendix 1), and spoken languages (English, Spanish, French, Portuguese), having a minimum experience of 3 years. All PTs completed cultural safety training, encompassing implicit bias, cultural awareness and humility, motivational interview training and strategies to build rapport with patients from diverse backgrounds. During onboarding, patients had a synchronous video call with their PT for an average duration of 30-60 minutes. During this call, the PT collected further medical history, conducted a clinical assessment, established goals through shared decision-making, and defined a tailored program following a patient-centered approach. Subsequent interactions between the patient and the PT were mostly asynchronous, where the therapeutic relationship was fostered through bidirectional communication via a secure chat within a smartphone app (Figure 1). Nonetheless, additional video calls were scheduled, whenever deemed appropriate by either patients or PTs.

Patients received a Food and Drug Administration (FDA)–listed class II medical device composed of a dedicated tablet with a mobile app, motion tracking, and a cloud-based portal. The DCP was conducted autonomously and asynchronously at the patient’s convenience, with exercise sessions being displayed on the tablet through videos with real-time audio and visual biofeedback on performance. The PT monitored and adjusted the program asynchronously through the cloud-based portal. Education covering pathophysiology, pain reconceptualization, active coping skills, the role of exercise, and fear-avoidance behaviors [26-28] was provided through short articles or videos via a smartphone app. The CBT component, delivered through interactive and audio modules via email, focused on mindfulness, acceptance and commitment therapy, and empathy-focused therapy [29].

### Outcome Measures

Patient engagement was evaluated by the completion rate, defined as the percentage of patients who completed the DCP, and by communication, estimated through the number of text interactions between patients and PTs.

Program satisfaction was assessed through the question “On a scale from 0 to 10, how likely is it that you would recommend this intervention to a friend or neighbor?”, recorded at the last reassessment. Clinical outcome data were collected at baseline and at the 9th, 18th, and 27th sessions as long as participants reached that milestone.

Clinical outcomes were analyzed for participants who reached the program end (ie, completers), where the last-available assessment data were used to determine the minimum clinically important change (MCIC), as described in Table 1.

**Table 1:** Clinical outcomes and corresponding patient-reported outcome measures with MCIC^a^ thresholds.

Response rate	Patient-reported outcome measure	MCIC threshold
Pain	The NPRS^b^ specific to the symptomatic body region: “Please rate your average pain over the last 7 days, from 0 (no pain at all) to 10 (worst pain imaginable).”	Improvement in pain by at least 30% according to IMMPACT (Initiative on Methods, Measurement, and Pain Assessment in Clinical Trials) guidelines [30]
Daily activity impairment	WPAI^c^ questionnaire: General Health v2.0 (range 0-100) [31]	Reduction of ≥9.1 [23]
Anxiety	GAD-7^d^ (range 0-21) [32,33]	Reduction of ≥3.8 [34]
Depression	PHQ-9^e^ (range 0-27) [33,35]	Reduction of ≥5 [36]

^a^MCIC: minimum clinically important change.

^b^NPRS: Numerical Pain Rating Scale.

^c^WPAI: Work Productivity and Activity Impairment.

^d^GAD-7: Generalized Anxiety Disorder 7-item scale.

^e^PHQ-9: Patient Health Questionnaire 9-item.

### Statistical Analysis

Descriptive statistics were reported using means and proportions. Comparisons between concordant and discordant groups were performed using independent-sample *t* tests or Mann-Whitney *U* tests for continuous variables (with Bonferroni correction) or chi-squared tests for categorical variables. Completion and clinical outcomes were binary variables, while satisfaction was a continuous variable. Two predictive variables describing concordance on race/ethnicity or on gender were created. Concordance was defined as an exact match on race/ethnicity between the patient and the PT, or a match on gender, depending on the variable of interest. Response rates were calculated using the last patient reassessment. In the case of missing reassessment at the discharge time point, the last available reassessment was carried forward.

Due to variability in the distribution of members allocated to each PT, a consequence of some PTs having been in the study longer, using PTs as random effects was not feasible. Generalized linear or logistic regression models were used to assess the effects of concordance on both race/ethnicity and gender on the described outcomes. Models of race/ethnicity concordance included an interaction term between concordance and patient race/ethnicity to explore the effect of concordance on outcomes stratified by race/ethnicity. Similarly, models of gender concordance included an interaction term between concordance and patient gender to explore the effect of concordance on outcomes stratified by gender. Given the baseline differences observed between groups in variables previously reported as predictors of recovery in patients with MSK conditions after physical therapy [37-39], all models were adjusted for the BMI (body mass index); age; education levels (dichotomized as low [no college degree] or high [at least a bachelor’s degree]); employment status (dichotomized as employed either full- or part-time or not currently employed); rurality; acuity (acute: pain<3 months; chronic: pain≥3 months) [40]); and baseline levels of pain, anxiety, and depression. Models assessing clinical outcomes were also adjusted for the discharged time point. A false discovery rate was used to adjust for multiple comparisons, and statistical significance was set at *P*<.05. Descriptive statistics were performed using commercially available software (IBM SPSS v22), and statistical analyses were performed using R Studio version 2023.09.1+494 (R Foundation for Statistical Computing).

### Ethical Considerations

All research was performed in accordance with relevant guidelines and regulations set by the Declaration of Helsinki. The original studies were prospectively approved by the New England (120190313) and Advarra (Pro00063337) independent Institutional Review Boards (IRBs) and registered on ClinicalTrials (NCT04092946, NCT05417685) on September 17, 2019, and June 14, 2022, respectively. All patients provided informed consent. Both IRB approvals and respective original consent covered secondary analysis without need for additional consent.

Prior to data analysis, all collected data underwent a rigorous anonymization process to safeguard the privacy of participants involved in the research. All individuals whose images are included in this publication (Figure 1) provided explicit written consent for the display of identifiable features in this manuscript, in accordance with journal guidelines and ethical standards. All data collection and analysis methods complied with relevant guidelines and regulations. Participants were not provided with any form of compensation.

## Results

### Participant Details

**Figure 2:**
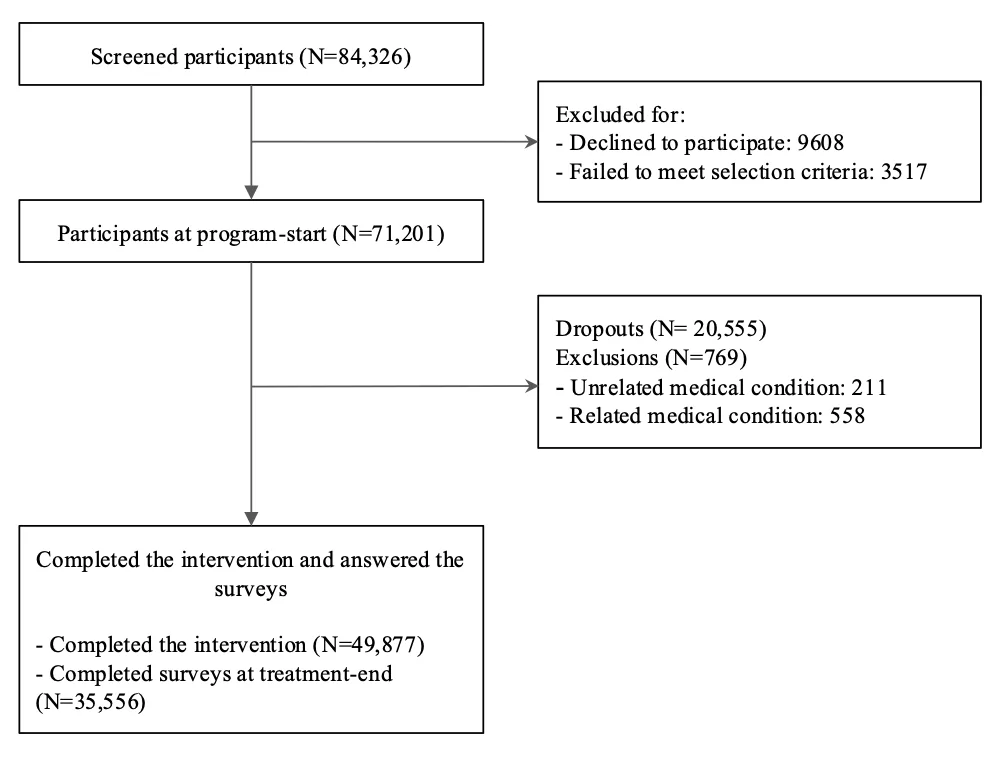
Study flowchart.

Of 84,326 eligible participants, 71,201 (84.4%) started the program, of which 49,877 (70.1%) completed the intervention (Figure 2). Of those who started the program, 63.9% (n=45,507) matched their PT’s race/ethnicity, while 61.2% (n=43,560) shared the same gender. Patients’ demographic and clinical characteristics stratified by race/ethnicity and gender concordance are reported in Table S2 in Multimedia Appendix 1. Race/ethnicity and gender distributions of the PTs (Figure S1 in Multimedia Appendix 1) were similar to the membership reported by the American Physical Therapy Association (APTA). The cohort was predominantly composed of middle-aged (mean 48.7 years, SD 11.8) women (n=42,464, 59.6%), who identified as White (n=49,920, 70.1%) and had a BMI above 30 (n=28,337, 39.9%). These individuals tended to have a high level of education (n=45,483, 63.8%), lived in urban locations (n=62,405, 87.6%), and reported full-time employment (n=61,298, 86.1%).

Regarding race/ethnicity, concordant and discordant groups were different in some demographic characteristics, such as age (mean 46.9, SD 11.5, vs mean 49.7, SD 11.8; *P*<.001), employment status (employed: n=22,898, 89.1%, vs n=38,400, 84.4%; *P*<.001), and rurality (n=1761, 6.9%, vs n=6708, 14.7%; *P*<.001). Differences were also observed among baseline clinical data, namely acuity (chronic: n=19,461, 75.9%, vs n=36,013, 79.3%; *P*<.001) and pain (mean 4.9, SD 2.0, vs mean 4.7, SD 1.9; *P*<.001).

Regarding gender, the concordant group had a larger proportion of women (n=33,854, 77.7%, vs n=8610, 31.1%; *P*<.001), patients working part-time (n=2215, 5.1%, vs n=770, 2.8%; *P*<.001), and patients with depression (mean 9.6, SD 4.3, vs mean 9.4, SD 4.2; *P*=.004) and daily activity impairment (mean 37.2, SD 22.8, vs mean 35.8, SD 22.4; *P*<.001).

### Engagement Outcomes

**Figure 3:**
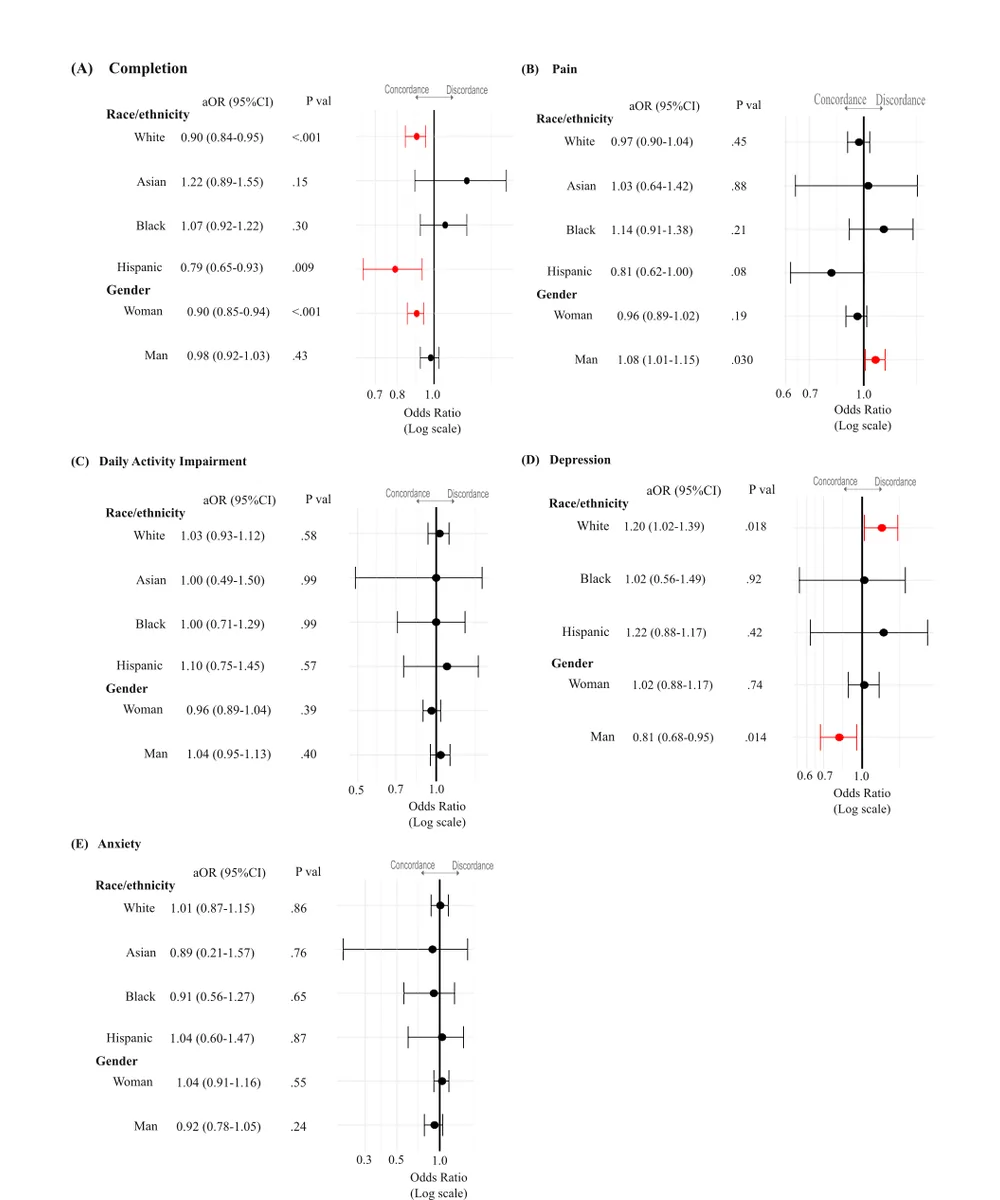
The aORs for concordance stratified by patient race/ethnicity and gender (reference: PT-patient concordance) for the following outcomes: (A) completion rate, (B) pain response rate, (C) daily activity response rate, (D) depression response rate; and (E) anxiety response rate. Statistically significant aORs are depicted in red. aOR: adjusted odds ratio.

The effect of race/ethnicity and gender concordance/discordance on completion rates and PT-patient interactions is presented in Figure 3 and Table 2, respectively. Both race/ethnicity and gender concordance were positively associated with the completion rate when compared to discordant dyads, particularly for those who identified as White (adjusted odds ratio [aOR] 1.11, 95% CI 1.05-1.19, *P*<.001), Hispanic (aOR 1.27, 95% CI 1.08-1.54, *P*=.009), and women (aOR 1.10, 95% CI 1.06-1.18, *P*<.001), as shown in Figure 3. PT-patient interactions were consistently high across the different race and gender dyads. Although there was a statistically significant difference between concordant and discordant dyads, this may not represent a clinically significant difference, as this variation ranged from 1 to 3 text interactions. An exception was observed among Asian patients, where a wider difference was observed, with discordant dyads reporting significantly higher interactions (adjusted β coefficient 5.32, 95% CI 3.28-7.36) compared to concordant dyads.

**Table 2:** Generalized linear model estimates of text interactions between patients and PTs^a^ stratified by patient race/ethnicity and gender (reference: PT-patient concordance).

Variable		Concordance, mean (95% CI); n (%)	Discordance, mean (95% CI); n (%)	Adjusted β coefficient (95% CI)	*P*-value^b^
**Race/ethnicity**					
	White	22.76 (22.13 to 23.39); 43,523 (87.2)	20.10 (19.39 to 20.80); 6397 (12.8)	–2.66 (–3.03 to –2.30)	<.001
	Asian	16.76 (14.65 to 18.87); 254 (3.6)	22.08 (21.37 to 22.78); 6773 (96.4)	5.32 (3.28 to 7.36)	<.001
	Black	20.17 (19.12 to 21.23); 1061 (14.4)	21.53 (20.82 to 22.24); 6284 (85.6)	1.36 (0.44 to 2.28)	.004
	Hispanic	19.48 (18.31 to 20.65); 669 (9.7)	21.20 (20.50 to 21.91); 6240 (90.3)	1.72 (0.67 to 2.77)	.001
**Gender**					
	Women	23.07 (22.44 to 23.70); 33,854 (79.7)	21.57 (20.89 to 22.25); 8610 (20.3)	–1.50 (–1.83 to –1.17)	<.001
	Men	20.60 (19.93 to 21.27); 9706 (66.2)	21.44 (20.80 to 22.09); 19,031 (33.8)	0.84 (0.51 to 1.17)	<.001

^a^PT: physical therapist.

^b^All *P* values were significant.

### Satisfaction

Program satisfaction was high across different races/ethnicities (mean 8.52, 95% CI 8.27-8.77, to mean 9.37, 95% CI 9.18-9.55) regardless of concordance status (all *P*>.05; Table 3), except for Asian patients who were statistically more likely to report higher satisfaction when matched in discordant dyads (adjusted β coefficient 0.36, 95% CI 0.12-0.59, *P*=.003; reference: concordance). Men showed a statistically higher likelihood to report satisfaction when matched in discordant dyads (adjusted β coefficient 0.15, 95% CI 0.10-0.20, *P*<.001; reference: concordance). In both cases (Asian patients and men), differences were small and therefore might not be clinically significant.

**Table 3:** Generalized linear model estimates of satisfaction level by patient race/ethnicity and gender (reference: PT^a^-patient concordance).

Variable		Concordance, mean (95% CI); n (%)	Discordance, mean (95% CI); n (%)	Adjusted β coefficient (95% CI)	*P*-value
**Race/ethnicity**					
	White	8.93 (8.84 to 9.02); 43,523 (87.2)	8.88 (8.78 to 8.98); 6397 (12.8)	–0.04 (–0.09 to 0.01)	.08
	Asian	8.52 (8.27 to 8.77); 254 (3.6)	8.88 (8.78 to 8.98); 6773 (96.4)	0.36 (0.12 to 0.59)	.003^b^
	Black	9.17 (9.02 to 9.31); 1061 (14.4)	9.19 (9.09 to 9.29); 6284 (85.6)	0.02 (–0.11 to 0.15)	.76
	Hispanic	9.37 (9.18 to 9.55); 669 (9.7)	9.24 (9.14 to 9.34); 6240 (90.3)	–0.13 (–0.30 to 0.04)	.13
**Gender**					
	Women	9.06 (8.97 to 9.15); 33,854 (79.7)	9.07 (8.98 to 9.17); 8610 (20.3)	0.01 (–0.03 to 0.06)	.61
	Men	8.68 (8.58 to 8.77); 9706 (33.8)	8.82 (8.73 to 8.92); 19,031 (66.2)	0.15 (0.10 to 0.20)	<.001^b^

^a^PT: physical therapist.

^b^Significant *P* values.

### Clinical Outcomes

The impact of race/ethnicity and gender on clinical outcomes is presented in Figure 3 and Table S3 in Multimedia Appendix 1.

Significant and similar pain response rates (adjusted: 62.16%, 95% CI 52.45-70.99, to 68.88%, 95% CI 63.10-74.12) were observed across different races/ethnicities. Regarding gender, men reported a slightly higher response rate when supported by women PTs (discordant dyads) in relation to concordant dyads (aOR 1.08, 95% CI 1.01-1.15, *P*=.03). Alongside pain recovery, daily activity impairment followed a similar trend, with high and significant response rates across all races/ethnicities and genders (69.03%, 95% CI 56.94-78.98 to 74.46%, 95% CI 70.81-77.81) regardless of concordance (all *P*>.05; Figure 3). Anxiety was similarly not impacted by concordance. In contrast, White patients had a higher depression response rate when supported by PTs from other races/ethnicities (aOR 1.20, 95% CI 1.02-1.39; reference: concordance), while men supported by men PTs were more likely to report significant depression recovery than discordant dyads (aOR 1.23, 95% CI 1.05-1.47, *P*=.01; reference: discordance).

### Effect of Covariates on Outcomes

The effect of covariates on outcomes among concordant/discordant dyads is depicted in Figure S2 in Multimedia Appendix 1 for race/ethnicity and Figure S3 in Multimedia Appendix 1 for gender.

Several covariates had an effect on the completion rate in both analyses (race/ethnicity concordance and gender concordance). A higher BMI, pain, depression and anxiety levels, and lower education levels were associated with a lower completion rate. In contrast, older age and not being employed (unemployed or retired) were associated with a higher completion rate. A lower pain response rate was observed across those with a higher BMI, lower education, and higher mental health scores. Chronic conditions and not being employed were also associated with smaller improvements in clinical outcomes.

## Discussion

### Principal Findings

The findings of this study demonstrated that being concordant on race/ethnicity and gender did not translate into higher satisfaction or better clinical outcomes in asynchronous digital care, despite the positive effect on treatment completion. Concordant dyads were associated with higher completion rates when compared to their counterparts, particularly for White participants (aOR 1.11, 95% CI 1.05-1.19, *P*<.001), Hispanic participants (aOR 1.27, 95% CI 1.08-1.54, *P*=.009), and women (aOR 1.10, 95% CI 1.06-1.18, *P*<.001). Race and gender dyads had frequent interactions, ranging from 16.8 to 23.01, with a minimal difference between groups. An exception was observed among Asian discordant dyads, which exchanged an adjusted mean of 5.32 more texts (95% CI 3.28-7.36, *P*<.001) than concordant dyads.

Program satisfaction was high regardless of concordance status (>8.52, 95% CI 8.27-8.77). Race/ethnicity concordance did not impact clinical outcomes for pain, anxiety, and daily activity impairment, as all improved similarly (eg, pain response rates ranged from 62.16%, 95% CI 52.45-70.99, to 68.88%, 95% CI 63.10-74.12, across different race/ethnicity dyads). An exception was observed for depression, in which White patients experienced a higher success rate when matched with PTs from other races/ethnicities (aOR 1.20, 95% CI 1.02-1.39; reference: concordance). In terms of gender concordance, men had a slightly higher pain response rate in discordant dyads (aOR 1.08, 95% CI 1.01-1.15, *P*=.03; reference: concordance) and a higher depression response rate in concordant dyads (aOR 1.23, 95% CI 1.05-1.47, *P*=.01; reference: discordance).

### Comparison With the Literature

Interactions in a digital context have distinct characteristics as they are primarily facilitated through video calls and text messaging. These forms of communication have been shown to reduce fears of embarrassment and foster a sense of support [41,42], as providers are readily accessible. However, it remains unclear whether the demographic characteristics of providers, and their concordance with patients, significantly influence intervention outcomes. Studies evaluating the effect of race/ethnicity or gender concordance on outcomes have been mainly conducted in primary/outpatient care [7-9,43,44] and behavioral health [45-47], making this study the first of its kind in the context of remote digital health. Herein, we explored this association in a large cohort whose demographic characteristics matched the US general population [48], particularly those reporting MSK pain [49]. Analyses were adjusted for key baseline characteristics that differed statistically between concordant and discordant groups and were previously reported to influence outcomes [37-39]. This approach reduces bias and permits a clearer understanding of the effect of racial/ethnic and gender concordance on outcomes. Our findings showed that concordance does not have a uniformly significant impact across all race/ethnicity, gender, and outcomes, as previously reported [15,50]. Importantly, the differences noted in some variables did not form a sustained pattern that can be operationalized in clinical practice.

Herein, concordance seemed to support higher completion rates, particularly among White and Hispanic groups and among women. Albeit in a different setting, a previous study reported that race concordance is important for retention in psychology interventions in adolescents [47]. Points of similarity may help enhance therapeutic relationships, resulting in a more trusting patient, willing to commit to the therapeutic plan developed collaboratively [51,52]. For example, women patients may find it easier to trust women PTs, who in turn may be more likely to empathize with them, given their common backgrounds [53].

High interaction levels were observed across all race/ethnicity and gender dyads, which are suggestive of therapeutic alliance establishment regardless of concordance. Further investigation will allow us to fully understand which factors of asynchronous communication may contribute more significantly to rapport building and therapeutic alliance establishment.

Similarly, high satisfaction was reported across dyads but was not generally impacted by concordance. Asian patients also reported slightly higher satisfaction when paired in discordant dyads, although this difference was small (adjusted β coefficient 0.36, 95% CI 0.12-0.59, *P*=.003), suggesting that it may lack clinical significance. The reason underlying these findings is unclear but may be related to unmeasured confounders. One can speculate that the diversity within the U.S. Census “Asian” category, encompassing groups ranging from Koreans to Asian Indians, may not reflect the true cultural or linguistic heterogeneity of the group [50,54]. The literature evaluating the effect of racial concordance on satisfaction has also shown inconclusive and contradictory findings [15].

Race/ethnicity concordance was not associated with significant improvements in most clinical outcomes (pain, daily activity impairment, and anxiety). An exception was depression, in which PTs’ demographic characteristics may have helped foster better outcomes. White discordant dyads reported better depression response rates, suggesting that although shared identity may facilitate a trustworthy relationship and engagement with the treatment, it is not the prime determining factor in sustaining a significant clinical outcome [15]. Additional factors, in addition to provider background, can carry significant weight in determining outcomes. In studies examining outcome predictors across nearly all pain conditions and therapies, the risk factors for treatment failure almost entirely comprise patient-related variables, including psychiatric morbidities, socioeconomic factors, or other clinical characteristics (eg, secondary gain, obesity, and smoking) [55-57]. Although some of these variables are modifiable, many are not, and even for those which are, addressing them may be time-consuming, prohibitively expensive, and fraught with other roadblocks.

Herein, the clinical team consisted of experienced clinicians, many with subspecialty training, all trained in equity, cultural humility, and unconscious bias, which may have reduced any subtle effects of concordance to the point of being undetectable with our study design [58,59]. Following Centers for Disease Control and Prevention (CDC) recommendations, training in cultural safety and humility is vital for empowering clinicians to adapt to diverse cultural groups, mitigating implicit racial bias and imbalances in patient-provider relationships, and promoting patient-centered care [44,60,61]. This is especially important in the light of the scarcity of diversity among providers compared to the general population, which is reflected in the physical therapy workforce [62,63].

Regarding the effect of gender concordance on clinical outcomes, only depression was influenced by concordance, with men showing a greater recovery trajectory when matched with a man PT. The reasons behind this finding are unknown, and the literature evaluating the effect of gender concordance on depression and other psychiatric outcomes is scarce. Depression is often deeply rooted in emotional beliefs and experiences [64], where societal and cultural aspects might play a significant role and influence patient-provider interactions [52]. Men might have felt more open and less stigmatized to share and discuss mental health issues when treated by a PT sharing the same gender identity. Interestingly, in the context of psychotherapy, men seem to exhibit no preference for therapist gender [45]. This evidence suggests that preferences observed in one clinical field may not necessarily apply to others and that caution is needed when generalizing findings across different outcomes, settings, or specialties, as the effects of concordance may be unique to each outcome and specific context.

### Strengths and Limitations

This study contains several important strengths: (1) the topic itself—the effect of race/ethnicity and gender concordance on engagement and clinical outcomes in the context of digital health using a fully remote DCP, laying the foundation for future research on this issue in the context of digital health; (2) the large sample size and proportional representation of patients from minority backgrounds, addressing a limitation identified previously [7,65]; and (3) the inclusion of validated outcome measures, previously lacking in comparable literature [50].

This study also displays limitations that warrant discussion. Considering that this DCP was mostly asynchronous, the results may not be generalizable for typical in-person or synchronous telerehabilitation contexts, where the face-to-face interaction between PTs and members is expected to be higher. Although the analysis included a wide range of patient demographic and clinical characteristics, it did not account for other variables that influence a patient’s experience with the provider (eg, years of experience, subspecialty training, and personality traits and compatibility). Additional subjective measures, such as empathy and cultural competency, which are difficult to capture, may have also impacted outcomes. Another limitation is the heterogeneity within race/ethnicity categories, particularly among Asian patients (which includes widely diverse groups). Although we considered adjusting for the random effects of the PT, the high variability of patients allocated to each PT precluded this analysis and should be addressed in future studies. Additionally, although potential bias from missing data cannot be ruled out, using the last observation carried forward provides the most conservative results.

Future research should focus on the impact of communication channels, cadence, and duration on the therapeutic alliance, particularly when comparing different delivery models (eg, in-person physical therapy). Additional research should address the impact of training (cultural, biased, or otherwise) and other PT characteristics as potential predictors of recovery outcomes in physical therapy. Another area for investigation is the concept of intersectionality in concordance (eg, multiracial and lesbian, gay, bisexual, transgender, queer or questioning, intersex, asexual, and other [LGBTQIA+] identity). Understanding these aspects is crucial, given the current demographic trends in the US and world populations, and could lead to more personalized and effective care strategies.

### Conclusion

Race/ethnicity and gender concordance between patients and PTs translated into higher treatment completion but did not impact satisfaction. For some clinical outcomes, discordant dyads reported greater improvements, suggesting that addressing provider diversity shortages requires more than just matching race/ethnicity or gender. Yet, the findings of this study did not identify a consistent pattern, suggesting that PTs’ characteristics, namely cultural safety training, experience, subspecialty training, and linguistic ability, may significantly impact engagement and outcomes in diverse populations. Future interventions should focus on these aspects to better understand their potential in optimizing patient experience and outcomes for MSK conditions.

## Appendix

Multimedia Appendix 1 Table S1: STROBE (Strengthening the Reporting of Observational Studies in Epidemiology) reporting checklist. Supplementary. Table S2: Baseline characteristics for the overall cohort and stratified by race/ethnicity concordance. Figure S1: Comparison of distribution of physical therapists between the study clinical team and the American Physical Therapy Association (APTA) in terms of (A) race/ethnicity and (B) gender. The study clinical team demographics were obtained from employer records. Table S3: Generalized linear model analysis of clinical outcome estimates by patient race and gender (reference: physical therapist-patient concordance). Figure S2: Effect of covariates on the outcome within the race concordance/discordance analysis. Figure S3. Effect of covariates on the outcome within the gender concordance/discordance analysis.

Multimedia Appendix 2 General overview of the exercise prescription framework.

## Abbreviations

aOR: adjusted odds ratio
BMI: body mass index
CBT: cognitive behavioral therapy
DCP: digital care program
GAD-7: Generalized Anxiety Disorder 7-item scale
MCIC: minimum clinically important change
MSK: musculoskeletal
NPRS: Numerical Pain Rating Scale
PHQ-9: Patient Health Questionnaire 9-item
PT: physical therapist
WPAI: Work Productivity and Activity Impairment

## Data Availability

The data presented in this study are available upon request from the corresponding author. The data are not publicly available due to privacy restrictions.
