# Addendum: Exploring the Importance of Race and Gender Concordance Between Patients and Physical Therapists in Digital Rehabilitation for Musculoskeletal Conditions: Observational, Longitudinal Study

**DOI:** 10.2196/97501

**Published:** 2026-08-27

**Authors:** Anabela C Areias, Dora Janela, Maria Molinos, Virgílio Bento, Carolina Moreira, Vijay Yanamadala, Steven P Cohen, Fernando Dias Correia, Fabíola Costa

**Affiliations:** 1 Sword Health, Inc New York, NY United States; 2 Instituto de Ciências Biomédicas Abel Salazar Porto Portugal; 3 Department of Surgery, Frank H Netter School of Medicine, Quinnipiac University Hamden, CT United States; 4 Department of Neurosurgery, Hartford Healthcare Medical Group Westport, CT United States; 5 Northwestern Feinberg School of Medicine Chicago, IL United States; 6 Walter Reed National Military Medical Center, Uniformed Services University of the Health Sciences Bethesda, MD United States; 7 Neurology Department, Centro Hospitalar e Universitário do Porto Porto Portugal

In the publication “Exploring the Importance of Race and Gender Concordance Between Patients and Physical Therapists in Digital Rehabilitation for Musculoskeletal Conditions: Observational, Longitudinal Study” [1], an addendum has been prepared to provide additional details regarding the exercise intervention and further enhance clarity and transparency.

An illustrative summary of exercise prescription, alongside typical exercise dosage, adjustments, and progression principles, has been included as Multimedia Appendix 2 (included here as Multimedia Appendix 1). This appendix provides a generalized framework underlying exercise prescriptions, which were individually adapted based on the patient’s condition and progression. Additional descriptions of digital care program implementations in specific musculoskeletal populations are available in previous studies [2,3], cited in the Introduction and Methods of the original article, respectively. These additions do not affect the study findings or conclusions of the original publication.

## Appendix

Multimedia Appendix 1 General overview of the exercise prescription framework.
