# Supplementary material for: Exploring the Importance of Race and Gender Concordance Between Patients and Physical Therapists in Digital Rehabilitation for Musculoskeletal Conditions: Observational, Longitudinal Study
Source: J Med Internet Res. 2024 Oct 29;26:e65354. doi: 10.2196/65354 (PMC11558217; doi:10.2196/65354)

MULTIMEDIA APPENDIX 1

Table S1. STROBE reporting checklist.

	Item No	Recommendation	Page No
**Title and abstract**	1	(*a*) Indicate the study's design with a commonly used term in the title or the abstract<br>(*b*) Provide in the abstract an informative and balanced summary of what was done and what was found	1-3
**Introduction**			
Background/rationale	2	Explain the scientific background and rationale for the investigation being reported	4-5
Objectives	3	State specific objectives, including any prespecified hypotheses	5
**Methods**			
Study design	4	Present key elements of study design early in the paper	5
Setting	5	Describe the setting, locations, and relevant dates, including periods of recruitment, exposure, follow-up, and data collection	5-6
Participants	6	(*a*) Give the eligibility criteria, and the sources and methods of selection of participants. Describe methods of follow-up<br>(*b*) For matched studies, give matching criteria and number of exposed and unexposed	6-7
Variables	7	Clearly define all outcomes, exposures, predictors, potential confounders, and effect modifiers. Give diagnostic criteria, if applicable	7-8
Data sources/ measurement	8*	For each variable of interest, give sources of data and details of methods of assessment (measurement). Describe comparability of assessment methods if there is more than one group	8
Bias	9	Describe any efforts to address potential sources of bias	10
Study size	10	Explain how the study size was arrived at	-
Quantitative variables	11	Explain how quantitative variables were handled in the analyses. If applicable, describe which groupings were chosen and why	8-9, Table 1
Statistical methods	12	(*a*) Describe all statistical methods, including those used to control for confounding<br>(*b*) Describe any methods used to examine subgroups and interactions<br>(*c*) Explain how missing data were addressed<br>(*d*) If applicable, explain how loss to follow-up was addressed<br>(*e*) Describe any sensitivity analyses	9-10
**Results**			
Participants	13*	(a) Report numbers of individuals at each stage of study—eg numbers potentially eligible, examined for eligibility, confirmed eligible, included in the study, completing follow-up, and analysed<br>(b) Give reasons for non-participation at each stage<br>(c) Consider use of a flow diagram	Figure 2 and Page 11

Descriptive data	14*	(a) Give characteristics of study participants (eg demographic, clinical, social) and information on exposures and potential confounders<br>(b) Indicate number of participants with missing data for each variable of interest<br>(c) Summarise follow-up time (eg, average and total amount)	Supplementary Table S2 and Table 2 and Table 3 & Supplementary Table S3, Pages 11
Outcome data	15*	Report numbers of outcome events or summary measures over time	Table 2 and Table 3 and Supplementary Table S3 and Pages 11
Main results	16	(*a*) Give unadjusted estimates and, if applicable, confounder-adjusted estimates and their precision (eg, 95% confidence interval). Make clear which confounders were adjusted for and why they were included<br>(*b*) Report category boundaries when continuous variables were categorized<br>(*c*) If relevant, consider translating estimates of relative risk into absolute risk for a meaningful time period	12-15
Other analyses	17	Report other analyses done—eg analyses of subgroups and interactions, and sensitivity analyses	-
**Discussion**			
Key results	18	Summarise key results with reference to study objectives	16
Limitations	19	Discuss limitations of the study, taking into account sources of potential bias or imprecision. Discuss both direction and magnitude of any potential bias	20
Interpretation	20	Give a cautious overall interpretation of results considering objectives, limitations, multiplicity of analyses, results from similar studies, and other relevant evidence	17-20
Generalisability	21	Discuss the generalisability (external validity) of the study results	20
**Other information**			
Funding	22	Give the source of funding and the role of the funders for the present study and, if applicable, for the original study on which the present article is based	22

*Give information separately for exposed and unexposed groups.

Note: An Explanation and Elaboration article discusses each checklist item and gives methodological background and published examples of transparent reporting. The STROBE checklist is best used in conjunction with this article (freely available on the Web sites of PLoS Medicine at http://www.plosmedicine.org/, Annals of Internal Medicine at http://www.annals.org/, and Epidemiology at http://www.epidem.com/). Information on the STROBE Initiative is available at http://www.strobe-statement.org.

Table S2. Baseline characteristics for the overall cohort and stratified by race/ethnicity concordance.

	Total Cohort (N=71,201)	Race/ethnicity		P-value	Gender		*P-value*
		Discordance (N=25,694)	Concordance (N=45,507)		Discordance (N=27,641)	Concordance (N=43,560)	
**Age (years), mean (SD)[a]**	48.7 (11.8)	46.9 (11.5)	49.7 (11.8)	*<.001*	49.0 (11.8)	48.5 (11.8)	*<.001*
**Age categories (years), N (%)[a]:**							
<25	1042 (1.5)	392 (1.5)	640 (1.4)	*<.001*	309 (1.1)	733 (1.7)	*<.001*
25-40	17,888 (25.2)	7635 (29.7)	10,253 (22.6)		6857 (24.8)	11,031 (25.4)	
41-60	39,551 (55.6)	14,269 (55.6)	25,282 (55.6)		15,230 (55.2)	24,321 (55.9)	
>60	12,618 (17.7)	3369 (13.1)	9249 (20.4)		5206 (18.9)	7412 (17.0)	
**Gender, N (%):**							
Women	42,464 (59.6)	15,044 (58.6)	27,420 (60.3)	*<.001*	8610 (31.1)	33,854 (77.7)	*<.001*
Men	28,737 (40.4)	10,650 (41.4)	18,087 (39.7)		19,031 (68.9)	9706 (22.3)	
**BMI (kg/m^2), mean (SD)[b]**	29.7 (7.1)	29.4 (7.1)	29.8 (7.1)	*<.001*	29.4 (6.5)	29.9 (7.4)	*<.001*
**BMI categories (kg/m^2), N (%)[b]:**							
Underweight (<18.5)	590 (0.8)	247 (1.0)	343 (0.8)	*<.001*	179 (0.6)	411 (0.9)	*<.001*
Normal (18.5-25)	18,731 (26.4)	7100 (27.7)	11,631 (0.8)		6745 (24.5)	11,986 (27.6)	
Overweight (≥25-30)	23,366 (32.9)	8509 (33.2)	14,857 (32.7)		10,194 (37.0)	13,172 (30.3)	
Obese (≥30-40)	22,162 (31.2)	7643 (29.8)	14,519 (32.0)		8544 (31.0)	13,618 (31.3)	
Morbidly Obese (≥40)	6175 (8.7)	2135 (8.3)	4040 (8.9)		1904 (6.9)	4271 (9.8)	

**Race/ethnicity, N (%):**							
Asian	7027 (99)	6773 (26.4)	254 (0.6)	*<.001*	3008 (10.9)	4019 (9.2)	*<.001*
Black	7345 (10.3)	6284 (24.5)	1061 (2.3)		2684 (9.7)	4661 (10.7)	
Hispanic	6909 (9.7)	6240 (24.3)	669 (1.5)		2872 (10.4)	4037 (9.3)	
White	49,920 (70.1)	6397 (24.9)	43,523 (95.6)		19,077 (69.0)	30,843 (70.8)	
**Education level, N (%):**							
Less than high school diploma	681 (1.0)	320 (1.2)	361 (0.8)	*<.001*	265 (1.0)	416 (1.0)	*<.001*
High school diploma	6652 (9.3)	2371 (9.2)	4281 (9.4)		2742 (9.9)	3910 (9.0)	
Some college	18,385 (25.8)	6396 (24.9)	11,989 (26.3)		6925 (25.1)	11,460 (26.3)	
Bachelor's degree	27,302 (38.3)	9671 (37.6)	17,631 (38.7)		10,674 (38.6)	16,628 (38.2)	
Graduate degree	18,181 (25.5)	6936 (27.0)	11,245 (24.7)		7035 (25.5)	11,146 (25.6)	
**Geographic location, N (%):**							
Urban	62,405 (87.6)	23,825 (92.7)	38,580 (84.8)	*<.001*	24,425 (88.4)	37,980 (87.2)	*<.001*
Rural	8469 (11.9)	1761 (6.9)	6708 (14.7)		3085 (11.2)	5384 (12.4)	
Not Available	327 (0.5)	108 (0.4)	219 (0.5)		131 (0.5)	196 (0.4)	
**Employment status, N (%):**							
Full-time job	61,298 (86.1)	22,898 (89.1)	38,400 (84.4)	*<.001*	24,559 (88.8)	36,739 (84.3)	*<.001*
Part-time job	2985 (4.2)	777 (3.0)	2208 (5.5)		770 (2.8)	2215 (5.1)	
Retired	3282 (4.6)	787 (3.1)	2495 (5.5)		1268 (4.6)	2014 (4.6)	
Not employed	2957 (4.2)	980 (3.8)	1977 (4.3)		808 (2.9)	2149 (4.9)	
Not Available/Prefers not to Answer	679 (1.0)	252 (1.0)	427 (0.9)		236 (0.9)	443 (1.0)	

Clinical data, mean (SD)							
**Symptomatic anatomical area, N (%):**							
Ankle	4562 (6.4)	1688 (6.6)	2874 (6.3)	*<.001*	1782 (6.4)	2780 (6.4)	*<.001*
Elbow	1962 (2.8)	666 (2.6)	1296 (2.8)		900 (3.3)	1062 (2.4)	
Hip	7719 (10.8)	2316 (9.0)	5403 (11.9)		2548 (9.2)	5171 (11.9)	
Knee	10,660 (15.0)	4057 (15.8)	6603 (14.5)		4123 (14.9)	6537 (15.0)	
Low back	24,212 (34.0)	8948 (34.8)	15,264 (33.5)		9721 (35.2)	14,491 (33.3)	
Neck	7408 (10.4)	2519 (9.8)	4889 (10.7)		2596 (9.4)	4812 (11.0)	
Shoulder	11,367 (16.0)	4153 (16.2)	7214 (15.9)		4680 (16.9)	6687 (15.4)	
Wrist	3072 (4.3)	1249 (4.9)	1823 (4.0)		1207 (4.4)	1865 (4.3)	
Other	239 (0.3)	98 (0.4)	141 (0.3)		84 (0.3)	155 (0.4)	
**Acuity, N (%)[c]:**							
Acute	15,608 (22.0)	6192 (24.1)	9416 (20.7)	*<.001*	6206 (22.5)	9402 (21.6)	*.007*
Chronic	55,474 (78.0)	19,461 (75.9)	36,013 (79.3)		21,391 (77.5)	34,083 (78.4)	
**Pain Intensity, mean (SD)**	4.8 (2.0)	4.9 (2.0)	4.7 (1.9)	*<.001*	4.7 (2.0)	4.8 (2.0)	*<.001*
**Mental health ≥5, mean (SD):**							
GAD-7[d]	8.7 (4.0)	8.8 (4.0)	8.7 (3.9)	.11	8.7 (3.9)	8.7 (4.0)	.08
PHQ-5[e]	9.5 (4.3)	9.5 (4.3)	9.5 (4.2)	.79	9.4 (4.2)	9.6 (4.3)	*.004*
**WPAI activities >0, mean (SD)[f]**	36.7 (22.7)	36.9 (23.1)	36.6 (22.5)	*<.001*	35.8 (22.4)	37.2 (22.8)	*<.001*

Note: Data represents mean (standard deviation) or the number of patients (% of total) where listed. Filtered cases correspond to participants who presented with clinically relevant baseline scores (eg, ≥5 in mental health variables). Sample size for: a: N=71,099; b: N=71,024; c: N=71,082; d: N= 24,002; e: N=71,186; f: N=53,954.

Figure S1. Comparison of Distribution of Physical therapists between the study clinical team and American Physical Therapy Association (APTA) in terms of (a) race/ethnicity; (b) gender. The study clinical team demographics were obtained from employer records.

(A)

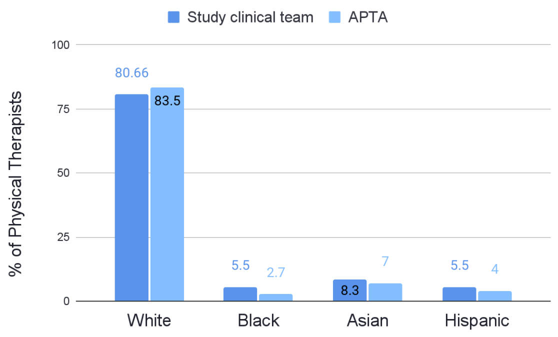

(B)

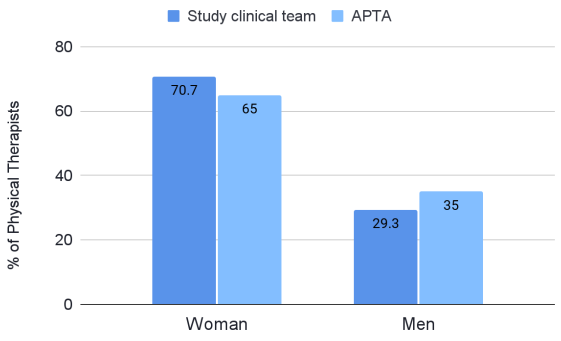

Table S3. Generalized linear model analysis of the clinical outcomes estimates by patient race and gender (reference = PT-patient concordance).

Variable	Concordance	Discordance	Odds Ratio (95% CI)	P-value
**Pain response rate**				
**Race/ethnicity**				
White	65.30 (62.31; 68.16) 26,100	64.67 (61.32; 67.87) 3654	0.97 (0.90; 1.04)	0.453
Asian	62.16 (52.45; 70.99) 119	62.84 (59.38; 66.18) 3641	1.03 (0.64; 1.42)	0.882
Black	62.68 (57.14; 67.92) 446	65.74 (62.32; 69.01) 2951	1.14 (0.91; 1.38)	0.208
Hispanic	68.88 (63.10;74.12) 361	64.26 (60.79; 67.58) 3025	0.81 (0.62; 1.00)	0.084
**Gender**				
Women	64.3 (61.3; 67.2) 19,278	63.3 (60.0; 66.5) 4761	0.96 (0.89; 1.02)	0.190
Men	65.6 (62.4; 68.7) 5505	67.3 (64.3; 70.2) 10,753	1.08 (1.01; 1.15)	*0.030*
**Depression response rate**				
**Race/ethnicity**				
White	42.4 (36.0; 49.0) 5174	46.9 (39.7; 54.3) 789	1.20 (1.02; 1.39)	*0.018*
Asian	60.0 (33.1; 81.9) 14	49.5 (41.6; 57.4) 514	ND*	ND*
Black	45.4 (33.5; 57.8) 90	46.0 (38.5; 53.7) 619	1.02 (0.56; 1.49)	0.916
Hispanic	47.9 (34.9; 61.1) 74	52.9 (45.4; 60.4) 643	1.22 (0.62; 1.83)	0.422
**Gender**				
Women	44.3 (37.8; 51.0) 4240	44.9 (37.9; 52.0) 1043	1.02 (0.88; 1.17)	0.744
Men	48.2 (40.9; 55.6) 855	43.0 (36.4; 50.0) 1779	0.81 (0.68; 0.95)	*0.014*

**Anxiety response rate**				
**Race/ethnicity**				
White	41.4 (36.0; 46.9) 7395	41.7 (35.7; 47.9) 1079	1.01 (0.87; 1.15)	0.861
Asian	51.0 (32.3; 69.4) 30	48.1 (41.6; 54.7) 853	0.89 (0.21; 1.57)	0.763
Black	52.1 (41.6; 62.5) 126	49.8 (43.3; 56.4) 815	0.91 (0.56; 1.27)	0.646
Hispanic	47.3 (36.3; 58.5) 108	48.1 (41.8; 54.6) 976	1.04 (0.60; 1.47)	0.871
**Gender**				
Women	42.2 (36.8; 47.7) 6270	43.1 (37.2; 49.1) 1524	1.04 (0.91; 1.16)	0.546
Men	46.3 (40.1; 52.5) 1203	44.1 (38.4; 50.0) 2385	0.92 (0.78; 1.05)	0.238
**WPAI activities response rate**				
**Race/ethnicity**				
White	71.34 (68.15; 74.34) 19,125	71.86 (68.30; 75.17) 2754	1.03 (0.93; 1.12)	0.577
Asian	69.03 (56.94; 78.98) 76	68.98 (65.10; 72.62) 2362	1.00 (0.49; 1.50)	0.993
Black	74.44 (68.19; 79.82) 287	74.46 (70.81; 77.81) 1806	1.00 (0.71; 1.29)	0.992
Hispanic	71.05 (63.68; 77.45) 215	72.92 (69.24; 76.32) 1995	1.10 (0.75; 1.45)	0.565
**Gender**				
Women	71.53 (68.32; 74.54) 14,015	70.79 (67.28; 74.06) 3559	0.96 (0.89; 1.04)	0.385
Men	71.49 (68.00; 74.75) 3746	72.26 (68.99; 75.31) 7300	1.04 (0.95; 1.13)	0.402

Note: significant P-values are italicized. *Low sample size to perform the calculation.

Figure S2. Effect of covariates on the outcome within the RACE concordance/discordance analysis.

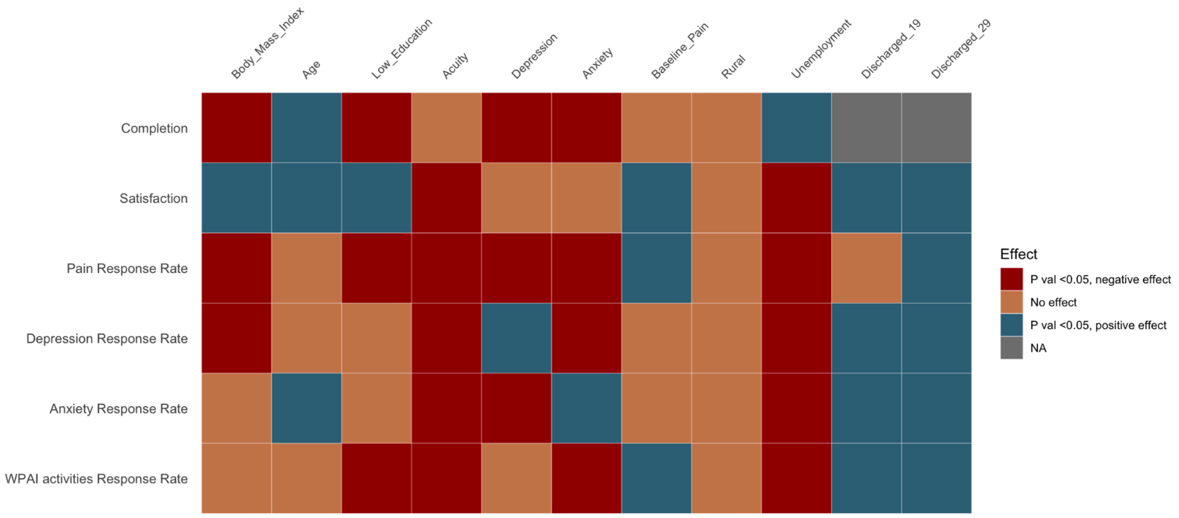

Figure S3. Effect of covariates on the outcome within the GENDER concordance/discordance analysis.

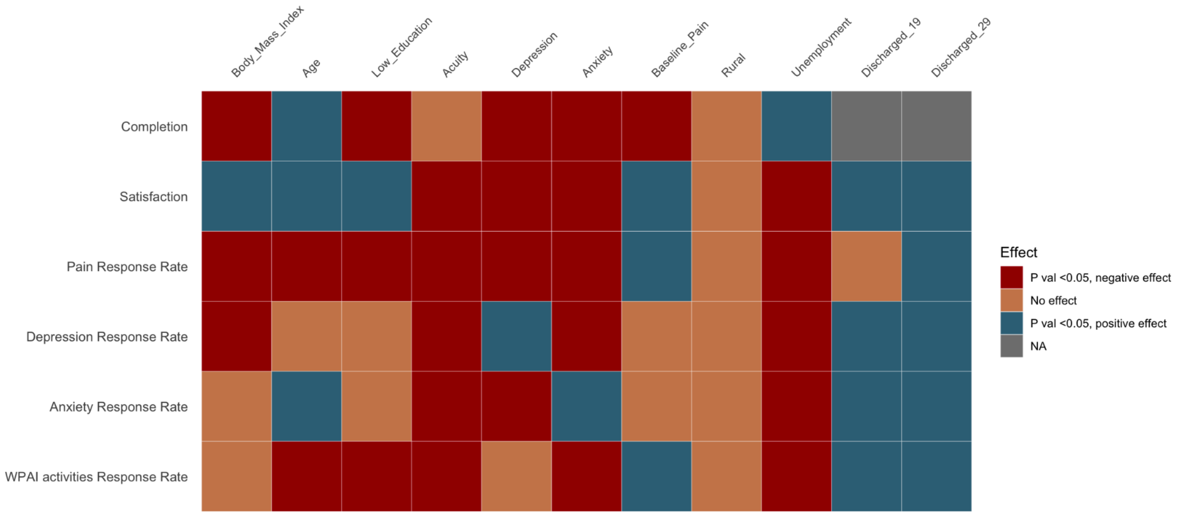

Supplement: Multimedia Appendix 1 [file jmir_v26i1e65354_app1.pdf]
