# Supplementary material for: Addendum: Exploring the Importance of Race and Gender Concordance Between Patients and Physical Therapists in Digital Rehabilitation for Musculoskeletal Conditions: Observational, Longitudinal Study
Source: J Med Internet Res. 2026 Aug 27;28:e97501. doi: 10.2196/97501 (PMC13563027; doi:10.2196/97501)
Supplement: Supplementary file 1 [file jmir_v28i1e97501_app1.docx]

**Table 1.** General overview of the exercise prescription framework.

| **Main Goals** | **Description** |
| --- | --- |
| **Initial Phase**  · Reduce symptom burden (e.g., pain, discomfort)  · Encourage gradual reintroduction of previously avoided movements  · Improve joint mobility and movement capacity  · Initiate low-load strengthening and motor control exercises | Programs typically included 1-2 sets per exercise of:  · Articular mobility exercises (e.g., hip external rotation; wrist flexion and extension)  · Strengthening exercises (e.g., sit to stand)  · Active and passive stretching exercises (e.g., neck side stretch)  · Condition-specific techniques when appropriate (e.g., ulnar nerve glides) |
| **Intermediate Phase**  · Further improve mobility and flexibility  · Progress muscular strength and endurance  · Increase tolerance to movement and activity-related symptoms  · Gradual reintroduction of functional and daily-life activities | Exercise was adjusted over time according to patient clinical presentation, typically by increasing the range of motion, adjusting resistance (e.g., increase external or bodyweight load), increasing exercise dosage (sets, repetitions, session duration) or increasing task complexity (e.g., stability demands).  Aim for at least 2-3 sets of:  · Articular mobility exercises (e.g., standing knee flexion)  · Strengthening exercises (e.g., lunges, glute bridges)  · Active and passive stretching exercises (e.g., pectoral stretch , hip adductors stretch) |
| **Late Phase**  · Restore functional capacity  · Return to usual daily and occupational activities  · Promote long-term self-management and independence | Add or increase exercise dosage (sets, repetitions, session duration) or load, and further increase task complexity (e.g., stability demands):  · Articular mobility exercises (e.g., single leg knee to chest)  · Strengthening exercises (e.g., split squats; standing overhead press, bent over row)  · Active and passive stretching exercises |

***Note:*** *Exercise prescription and progression was individualized based on baseline clinical assessment, ongoing feedback from the patient, performance metrics captured by the motion tracking technology during sessions, and symptom evolution (pain, fatigue, functional capacity). Typical Dosage (illustrative):*

*·	Recommended frequency: at least 3 sessions per week*

*·	Treatment duration: up to 12 weeks*

*·	Repetition ranges commonly varied (e.g., 8–15 reps depending on goal)*

*·	Programs adjusted dynamically based on tolerance and progression.*
